# Non-specific Detection of a Major Western Blotting Band in Human Brain Homogenates by a Multitude of Amyloid Precursor Protein Antibodies

**DOI:** 10.3389/fnagi.2019.00273

**Published:** 2019-10-09

**Authors:** Hazal Haytural, Jolanta L. Lundgren, Tansu B. Köse, Tomàs Jordà-Siquier, Marinela Kalcheva, Mohammed Seed Ahmed, Bengt Winblad, Erik Sundström, Gaël Barthet, Lars O. Tjernberg, Susanne Frykman

**Affiliations:** ^1^Division of Neurogeriatrics, Center for Alzheimer Research, Department of Neurobiology, Care Science and Society, Karolinska Institutet, Solna, Sweden; ^2^Interdisciplinary Institute of Neuroscience, Université de Bordeaux, Bordeaux, France; ^3^Wolfson Centre for Age-Related Diseases, King’s College London, London, United Kingdom

**Keywords:** amyloid precursor protein, human brain, western blotting, cross-reactivity, η-secretase

## Abstract

The use of human post-mortem brain material is of great value when investigating which pathological mechanisms occur in human brain, and to avoid translational problems which have for example been evident when translating animal research into Alzheimer disease (AD) clinical trials. The amyloid β (Aβ)-peptide, its amyloid precursor protein (APP) and the intermediate APP-c-terminal fragments (APP-CTFs) are all important players in AD pathogenesis. In order to elucidate which APP CTF that are the most common in brain tissue of different species and developmental stages, and whether there are any differences in these fragments between AD and control brain, we investigated the occurrence of these fragments using different APP c-terminal antibodies. We noticed that whereas the conventional APP-CTFα and CTFβ fragments were most prominent in rat and mouse brain tissue, the major western blotting band detected in human, macaque and guinea pig was of approximately 20 kDa in size, possibly corresponding to the newly discovered APP-CTFη. However, this band was also intensely stained with a total protein stain, as well as by several other antibodies. The staining intensity of the 20 kDa band by the APP antibodies varied considerably between samples and correlated with the staining intensity of this band by the total protein stain. This could potentially be due to non-specific binding of the antibodies to another protein of this size. In-gel digestion and mass spectrometry confirmed that small amounts of APP were present in this band, but many other proteins were identified as well. The major hit of the mass spectrometry analysis was myelin basic protein (MBP) and a myelin removal protocol removed proportionally more of the 20 kDa APP band than the full-length APP and APP-CTFα/β bands. However, the signal could not be immunodepleted with an MBP antibody. In summary, we report on a potentially non-specific western blotting band of approximately 20 kDa and call for precaution when analyzing proteins of this size in human brain tissue.

## Introduction

Alzheimer disease (AD) is the most common form of dementia and causes extensive suffering for millions of patients and their relatives. Unfortunately, the only medication available gives just subtle symptomatic relief, and a large number of clinical trials of disease-modifying drugs have failed in the last decade. This is partially due to problems with translation, i.e., compounds that work well in mouse models fail in clinical trials. Thus, it is of utmost importance to study the alterations in human AD brain.

The synaptotoxic amyloid β-peptide (Aβ) plays a critical role in the pathophysiology of AD (Selkoe, 2011; Prince et al., 2013). Aβ is generated by the cleavage of the 695–770 amino acid long amyloid precursor protein (APP) by first β-secretase and then the γ-secretase complex. After the first cleavage by β-site APP cleaving enzyme 1 (BACE1), the main β-secretase of neurons, the 99 or 89 amino acid long c-terminal fragment (CTFβ) remains in the membrane while the soluble APPβ (sAPPβ) is released. The CTFβ is further cleaved by the protein complex γ-secretase, generating Aβ and the APP intracellular domain (AICD; Selkoe, 2011). In a separate, non-amyloidogenic pathway, APP is cleaved by the α-secretase a disintegrin and metalloproteinase 10 (ADAM10) instead of BACE1. This cleavage produces the 83 amino acids long CTFα which can also be cleaved by the γ-secretase complex, thus precluding Aβ production since the α-cleavage site is situated within the Aβ sequence (Postina et al., 2004).

APP can also be cleaved by a number of other proteases, such as caspases (Galvan et al., 2002), aspargine endopeptidase (δ-secretase; Zhang et al., 2015), the recently identified η-secretase (Wang et al., 2015; Willem et al., 2015), or other unknown proteases (Nikolaev et al., 2009), to produce toxic fragments and/or influence further processing. The situation is further complicated by the fact that also CTFβ and the fragment derived from sequential cleavage by η- and α-secretase (Aη-α) have been shown to be neurotoxic and may therefore be involved in the pathology of AD (Oster-Granite et al., 1996; McPhie et al., 1997; Bittner et al., 2009; Jiang et al., 2010; Lauritzen et al., 2012; Willem et al., 2015). However, which of these fragments that are most abundant in human brain, and whether the processing is the same in different species, or at different developmental stages, has to our knowledge not been carefully investigated.

Here, we studied the occurrence of different APP-derived fragments in human as well as in other species including macaque, guinea pig, rat and mouse brain using western blotting. Using several APP-antibodies we detected a major band of approximately 20 kDa in adult human, macaque and guinea pig brain. However, more careful investigation using total protein stain, mass spectrometry and immunoprecipitation indicated that this band was unspecific and was also recognized by several other antibodies.

## Materials and Methods

### Human Post-mortem Material

The use of human brain material in this study was conformed to the Declaration of Helsinki and approved by the regional ethical review board of Stockholm (2015/1803-31/2 and 2007/1477-3). Brain tissues from frontal cortex Brodmann area 9 (BA9) of 10 sporadic AD and 10 control cases were obtained from Brains for Dementia Research, London, UK, while the human brain tissue (mixed cortex) used for the experiments with the different antibodies, immunoprecipitation and different species was obtained from the Brain Bank at Karolinska Institutet, Stockholm, Sweden. All patients were clinically and pathologically diagnosed and the characteristics of the patients are described in Table 1. Cortical brain tissue from human fetuses, post-conception age 7–11 weeks, was obtained from clinical routine abortions through the Developmental Tissue Bank at Karolinska Institutet. All participants or their consultees (for participants that lacked capacity) gave informed consent to the donations and all procedures were approved by ethical review boards.

**Table 1:** Characteristics of cases for post-mortem human brain samples Brodmann area 9 (BA9) obtained from the Brains for Dementia Research.

	Age (years)	Gender	PMD (h)
Sporadic AD (*n* = 10)	88.7 ± 6.6	70% F	34.0 ± 23.3
Control (*n* = 10)	82.3 ± 7.4	70% F	35.7 ± 20.2

*F, female; PMD, post-mortem delay*.

### Animals

Male Wistar rats (Charles River) were killed by carbon dioxide treatment while female C57BL/6 mice (bred at Karolinska Institutet) were killed by cervical dislocation. The animals used in this study were handled according to the Karolinska Institutet guidelines, Swedish national guidelines and current European Law (Directive 2010/63/EU). The use of rat brains was approved by the animal research ethical committee of southern Stockholm (S21-14) while the use of mouse brain was approved by Linköping ethical committee (ID156). Brain lysates from guinea pig and macaque was purchased from Novus Biologicals who ensure that the animals have been handled according to the United States Department of Agriculture (USDA) animal welfare act as well as the National Institutes of Health (NIH), Office of Laboratory Animal Welfare (OLAW) and the Public Health Service (PHS) policy on humane care and use of laboratory animals. No experiments were performed on live animals.

### Brain Homogenization

Brain homogenates derived from different sources were homogenized in slightly different buffers and with slightly different protocols. For all samples except the ones mentioned specifically below the following method was used: homogenization of the cortical tissue from human, rat and mouse was carried out in four volumes of cold homogenization buffer [20 mM HEPES, 150 mM NaCl, 5 mM ethylenediaminetetraacetic acid (EDTA), pH 7.0] with Complete Protease Inhibitor Cocktail (Roche) by eight strokes at 800 rpm using a mechanical glass-teflon homogenizer. Brain tissues from human and mouse embryo were then sonicated. AD and control samples from the Brains for Dementia Research were homogenized in 50 mM Tris-HCl, 5 mM EGTA, 10 mM EDTA and Sigma protease inhibitor cocktail. The guinea pig and macaque homogenates were delivered as ready lysates from Novus Biologicals. Protein determination was done by Pierce™ BCA Protein Assay Kit (Thermo Fisher). The brain homogenates were stored at −80°C.

### Antibodies

**Figure 1:**
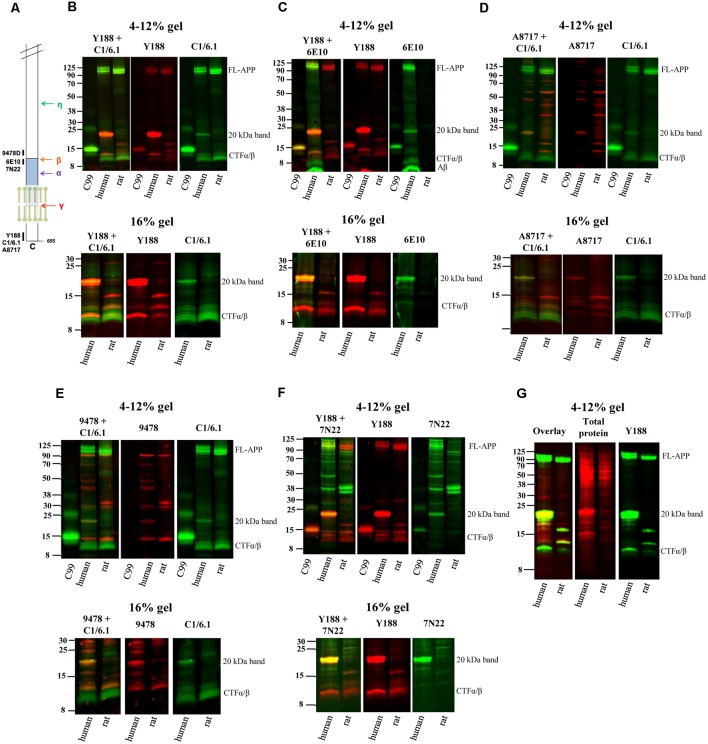
Elucidating the pattern of bands detected by different amyloid precursor protein (APP) antibodies in rat and human brain. **(A)** The APP protein, indicating the epitopes of the antibodies used in the study. **(B–F)** Equal amounts of homogenates of human Alzheimer disease (AD) and adult rat brain were loaded on 4%–12% (upper panels) or 16% (lower panels) sodium dodecyl sulfate polyacrylamide gel electrophoresis (SDS-PAGE) gels and subjected to western blotting using the Odyssey digital fluorescent system with detection by primary antibodies **(B)** Y188 and C1/6.1, **(C)** Y188 and 6E10, **(D)** A8717 and C1/6.1, **(E)** 9478 and C1/6.1 or **(F)** Y188 and 7N22. A recombinant C99-FLAG peptide was loaded on the 4%–12% gels to indicate the approximate position of c-terminal fragments (CTF)-β (minus the 1 kDa FLAG-tag). The figures are representative figures and four additional rat brain samples and five additional human brain samples showed a similar pattern using the Y188 and C1/6.1 antibodies (see also Figure 5). **(G)** Equal amounts of homogenates of human AD and adult rat brain were loaded on a 4%–12% SDS-PAGE gel and subjected to western blotting using the Odyssey digital fluorescent system with detection by primary antibody Y188 together with a total protein stain. A major band was detected around 20 kDa by all antibodies as well as by the total protein stain.

All primary antibodies used in the study are described in Table 2 and the epitopes of the APP antibodies are delineated in Figure 1A. The secondary antibodies IRDye 800CW donkey anti-mouse IgG and IRDye 680RD donkey anti-rabbit IgG were purchased from LI-COR^®^. For the in-gel digestion, we instead used a horseradish peroxidase (HRP)-conjugated goat anti-rabbit antibody (GE-Healthcare).

**Table 2:** List of primary antibodies.

Antibody name	Company and product number	Dilution (WB)	Final concentration (μg/ml)
Anti-amyloid beta precursor protein (Y188)	Abcam (ab32136)	1:5,000	0.08
Anti-APP c-terminal fragment (C1/6.1)	BioLegend (802801)	1:5,000	0.2
Anti-amyloid beta (6E10)	BioLegend (803001)	1:1,000	1
Anti-amyloid beta precursor protein (A8717)	Sigma (A8717)	1:2,000	5
Anti-beta amyloid (7N22)	Invitrogen (AHB0272)	1:5,000	0.1
Anti-amyloid beta precursor protein (9478)	Gift from Dr. Willem	1:500	4
Anti-myelin basic protein (MBP)	Abcam (ab216668)	1:500	0.1
Anti-kelch-like ECH-associated protein 1 (KEAP1)	Protein Tech (10503-2-AP)	1:5,000	0.07
Anti-vitamin D-binding protein (GC)	AbFrontier (LF-MA0147)	1:20,000	0.025

### SDS-PAGE and Western Blotting

Equal amounts of protein (30–40 μg depending on experiment) from each sample were denatured in either 4× of protein sample loading buffer (LI-COR) containing 2-mercaptoethanol, or 4× NuPAGE LDS sample buffer containing dithiothreitol (DTT), or 2× Laemmli buffer and boiled at 95°C for 5 min. Samples and 7 μl of the Chameleon duo pre-stained protein ladder (LI-COR) were separated by sodium dodecyl sulfate polyacrylamide gel electrophoresis (SDS-PAGE) on 4%–12% polyacrylamide bis-tris gels (Invitrogen) or 16% polyacrylamide tricine gels (Invitrogen) and transferred to nitrocellulose membranes (GE Healthcare). As a reference, a recombinant C99-FLAG peptide (a gift from Dainippon Sumitomo Pharma) was loaded. After transfer, nitrocellulose membranes were blocked in Odyssey blocking buffer (TBS) for up to 1 h at room temperature, followed by incubation with primary antibodies at 4°C overnight. Membranes were washed with TBS-T and then incubated with fluorescently labeled IRDye secondary antibodies (LI-COR) for 1 h at room temperature. After TBS-T washes, membranes were washed in TBS for one last time, and digital fluorescent visualization of signals was detected at 700 and/or 800 nm channels using the Odyssey^®^ CLx Imaging System (LI-COR). All primary and secondary antibodies were diluted in Odyssey blocking buffer (TBS). In some experiments, membranes were stained with a total protein stain [REVERT™ Total Protein Stain (LI-COR)] especially for normalization of the signal (Figures 3A,B). In order not to interfere with the signal that could arise from antibodies, this step was done right after transfer. Once membranes were incubated with total protein stain and the signal was immediately detected at the 700 nm channel. After removal of the stain using reversal buffer, the membrane was incubated with Odyssey blocking buffer (TBS) and incubation with primary and secondary antibodies was done as mentioned above. Quantitation of protein (20 kDa band and total protein) was done using Image Studio Lite v5.2 (LI-COR). In order to assure linearity of the signals three different concentrations of a standard sample (A1) was loaded on all gels and a standard curve was created which was used for quantification. Statistical analysis of the comparison of the levels of the 20 kDa band in AD and control was performed using Student’s *t*-test.

**Figure 2:**
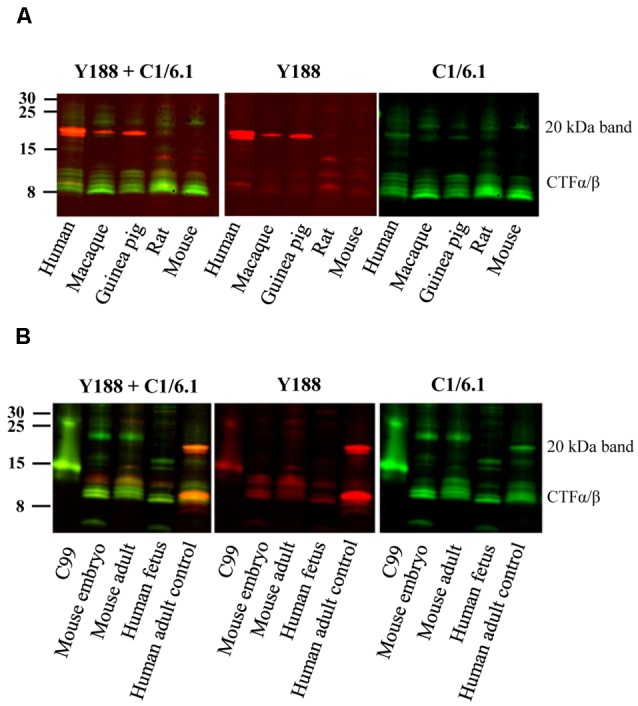
Bands detected by APP antibodies in different species and developmental stages. **(A)** Equal amounts of human AD, macaque, guinea pig, rat and mouse brain homogenates were loaded on a 16% gel and subjected to western blotting using the Y188 and the C1/6.1 antibodies. The major 20 kDa band is detected in human, macaque and guinea pig brain, but not in rat or mouse brain. **(B)** Equal amounts of brain homogenates from mouse embryo, adult mouse, human embryo and human adult control were loaded on a 16% gel. The Y188 and C1/6.1 antibodies were used for detection. The 20 kDa band was only detected in adult human brain whereas only lower molecular CTFs were present in human fetal brain. The pattern of CTFs was similar in embryonic and adult mouse brain. Panel **(B)** is a representative figure and the results were confirmed using an additional three human and four mouse embryonic brain samples.

**Figure 3:**
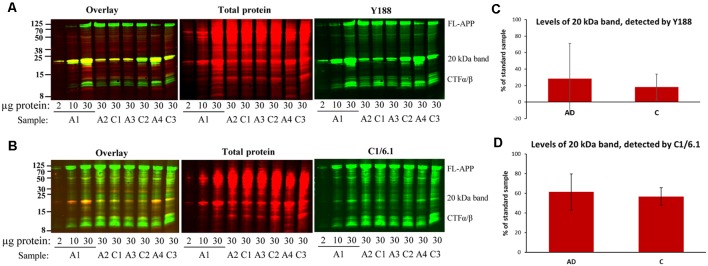
Bands detected by APP antibodies in human AD and control brain homogenates. Equal amounts of homogenates form AD and control brain were loaded on 16% gels. In order to assure linearity of the signals, three different concentrations of a standard sample (A1) was loaded on all gels and a standard curve was created which was used for quantification. The gels were subjected to SDS-PAGE and western blotting using **(A)** Y188 antibody together with a total protein stain or **(B)** C1/6.1 antibody together with a total protein stain. Representative gels are shown with age- and gender-matched pairs of AD and control cases loaded beside each other. **(C,D)** Densitometric analysis of the 20 kDa band using the Y188 **(C)** or the C1/6.1 antibody **(D)**. All values were normalized to the total protein stain and expressed as % of the standard sample. The major 20 kDa APP band varies considerably between cases and correlates with the size and intensity of a major total protein stained band. No difference in the levels of the 20 kDa band could be detected between AD and control brain. A, AD; C; control.

For the in-gel digestion experiment, equal amounts of samples were loaded onto the 4%–12% polyacrylamide bis-tris gels (Invitrogen) and transferred to polyvinylidene difluoride (PVDF) membranes (GE Healthcare). Following primary antibody incubation, membranes were incubated with secondary antibodies coupled to HRP (GE-Healthcare). SuperSignal West Pico enhanced chemiluminescent reagent (Pierce) was then used to visualize the signals which were exposed to film (GE-Healthcare).

### In-Gel Digestion and Mass Spectrometry

Protein bands were excised manually from a bis-tris 4%–12% gel, using western blotting as a location reference, and in-gel digested using a MassPREP robotic protein-handling system (Waters). Gel pieces were destained twice with 100 μl 50 mM ammonium bicarbonate (Ambic) containing 50% acetonitrile at 40°C for 10 min. Proteins were reduced with 10 mM DTT in 100 mM Ambic for 30 min at 40°C and alkylated with 55 mM iodoacetamide in 100 mM Ambic for 20 min at 40°C followed by in-gel digestion with 0.3 μg Trypsin (Sequence grade, Promega) in 50 mM Ambic for 5 h at 40°C. The tryptic peptides were extracted with 1% formic acid in 2% acetonitrile, followed by 50% acetonitrile twice. The liquid was evaporated to dryness and the peptides were separated on an EASY-spray column connected to an EASY-nLC 1000 system (Thermo Scientific). The peptides were eluted in a 60 min gradient (from 5% to 26% of buffer B (2% acetonitrile, 0.1% formic acid) in 55 min and up to 95% of buffer B in 5 min) at a flow rate of 300 nL/min and analyzed on a Fusion Orbitrap mass spectrometer (Thermo Scientific). The spectra were analyzed using the Mascot search engine v.2.4 (Matrix Science Limited).

### Immunoprecipitation

For immunoprecipitation with the Y188 and A8717 antibodies, antibodies or a rabbit IgG (used as a negative control) were bound to PureProteome™ Protein A/G mix magnetic beads (Millipore). Brain homogenates from AD cortex were solubilized in 1× RIPA lysis buffer without NP-40 (150 mM NaCl, 0.5% sodium deoxycholate, 0.1% SDS, 50 mM Tris/HCl, pH 8.0) containing Complete Protease Inhibitor Cocktail (Roche). The sample was precleared with magnetic beads for 2 h at 4°C in order to remove any potential non-specific binding. Twenty microliter of sample was collected as input for western blotting, whereas the remaining sample was incubated with the antibody coupled beads overnight at 4°C. After immunoprecipitation, 20 μl of sample was collected to detect unbound proteins. Precipitated proteins were washed with PBS and eluted in 20 μl of 1× NuPAGE LDS sample buffer (Invitrogen) and boiled at 95°C for 5 min. The eluates were transferred to new microcentrifuge tubes and subjected to western blotting as described above.

For myelin basic protein (MBP) immunodepletion, brain homogenate from AD cortex was solubilized in 1× RIPA lysis buffer as mentioned above, and the sample was precleared with magnetic beads for 30 min at room temperature in order to remove any potential non-specific binding. Twenty microliter of sample was collected as inputs before and after pre-absorption with beads, respectively. The remaining sample was incubated with the MBP antibody for 1 h at room temperature to create antibody-antigen complex, which was followed by incubation with the magnetic beads for 30 min at room temperature. After immunoprecipitation, 20 μl of the unbound sample was collected and the sample bound to the beads was washed three times in 1× RIPA. Precipitated proteins were eluted in 40 μl of 1× Protein Sample Loading buffer (LI-COR) and boiled at 95°C for 5 min. The eluates were transferred to new microcentrifuge tubes and subjected to western blotting as described above.

### Myelin Removal

Myelin Removal Beads II (Miltenyi Biotec) was used according to the manufacturer’s protocol with some modifications. The protocol builds on binding of myelin to a specific but confidential antibody coupled to the beads. Sixty microliter beads were added to 500 μl of AD brain homogenate in 0.5% BSA in PBS (1.5 μg/μl) and incubated for 15 min, rotating at room temperature. The sample was then further diluted 5.5 times with PBS/BSA and centrifuged at 100,000× *g*, 30 min and resuspended in 1 ml of PBS/BSA in order to first wash and then concentrate the membranes. The sample was then passed through a magnetic LS column and the flow-through (myelin-depleted sample) was collected.

## Results

### A Multitude of APP Antibodies Labels a 20 kDa Predominant Band in Human Brain

Using different APP antibodies, we analyzed APP fragments in human AD and rat brain homogenates after separation on BisTris 4%–12% polyacrylamide gels or tricine 16% polyacrylamide gels. The use of the LI-COR detection system, which allows simultaneous detection with two different antibodies, enabled us to visualize whether the same band was stained with the different antibodies. Surprisingly, we found that in human brain, a predominant band of approximately 20 kDa was detected by all antibodies tested, including Y188 (Figures 1B,C,F), C1/6.1 (Figures 1B,D,E), 6E10 (Figure 1C), A8717 (Figure 1D), 9478 (Figure 1E) and 7N22 (Figure 1F). From now on, we will refer to this band as the 20 kDa band although the molecular weight varies slightly depending on gel systems and molecular weight markers, and the exact size has not been determined. In contrast to human brain, the 20 kDa band was not detected in rat brain homogenates by any of the antibodies. The 20 kDa band corresponds to the expected weight of the proposed APP-CTFη, generated by η-secretase cleavage at N504 in the APP695 sequence (Willem et al., 2015). When using the Y188 antibody and 16% gels, which effectively separate proteins of lower molecular weight, we observed that the 20 kDa band is actually a double band (Figures 1B,C,F) in agreement with the double CTF-η band detected by Wang et al. (2015).

ADAM10 cleavage of APP gives rise to the 83 amino acids long CTFα, while CTFβ is either 99 or 89 amino acids long depending on where APP is cleaved by BACE1. In addition, post-translational modifications of APP, such as phosphorylation and glycosylation may give rise to even more CTF bands using western blotting. Thus, several bands between 8 and 14 kDa were detected, most clearly demonstrated with the C1/6.1 antibody. Using this antibody, the staining intensity for the CTFα and -β bands were similar between human and rat homogenates (Figures 1B,D,E). The 9478 antibody is directed to the N-terminal part of the CTFη sequence and is not expected to detect CTFα or -β. Instead, the band of approximately 12 kDa detected by 9478 could be the proposed Aα-η peptide (Figure 1E). A major human-specific band of around 11 kDa was detected with the Y188 antibody. On 16% gels, this band migrates as one of the lower conventional CTF bands (Figures 1B,C,F, lower panels). Since this band does not overlap with bands detected by 6E10, we can conclude that it does not include the first eight amino acids in the Aβ sequence and is thus not identical to C99. Y188 also detected a major 15 kDa fragment in rat brain, possibly reflecting CTFδ (Zhang et al., 2015).

In addition, the expected immature (non-glycosylated) and mature (glycosylated) forms of full-length APP [FL-APP, approximately 100 kDa (Weidemann et al., 1989)] were clearly detected with the Y188, C1/6.1 and 6E10 antibodies (Figures 1B–F), whereas the A8717 did not detect these bands (Figure 1D), 9478 detected a band of slightly smaller molecular weight (Figure 1E) and 7N22 only detected the lower FL-APP band in human brain (Figure 1F). In general, the A8717, 9478 and 7N22 antibodies detected a multitude of bands of unexpected molecular weight, possibly due to non-specific or cross-reactive binding. The staining intensity of FL-APP and APP-CTFα and -β were similar between human and rat when detected with the Y188 or C1/6.1 antibodies. Since 6E10 is a human-specific antibody that recognizes the first amino acids in the APP-CTFβ sequence, no bands were detected in rat brain homogenate using this antibody and the lower CTFα/β bands, corresponding to CTFα were not detected (Figure 1D).

Peculiarly, the ratio between the different western blotting bands varied between the different antibodies. Whereas the 20 kDa band gave rise to the strongest signal of all APP-derived fragments in human brain using the Y188 or 6E10 antibodies (Figures 1B,C,F), the signal from this band was about as intense as the signal from the other CTFs when using the C1/6.1 antibody (Figures 1B,D,E). However, the 20 kDa band was always stronger in human than in rat brain samples. We also noticed that a band stained intensively with a total protein stain, overlapped with the 20 kDa band (Figure 1G).

In summary, using six different APP antibodies (Y188, C1/6.1, 6E10, A8717, 9478 and 7N22), we detected a 20 kDa band in human AD brain homogenate which was hardly detectable in rat brain homogenate. In the subsequent experiments, we chose to focus on the combination of Y188 and C1/6.1 antibodies. It should also be noted that although all experiments in Figure 1 are performed using the same human brain homogenate, the 20 kDa band was present in almost all human brain samples tested (see Figure 5) while being non-detectable or present in very low levels in five additional rat brain samples (data not shown). The presence of the 20 kDa band was not dependent on sample treatment, since it was present independently on which loading buffer that was used (data not shown) or if the samples were treated with RIPA buffer.

**Figure 4:**
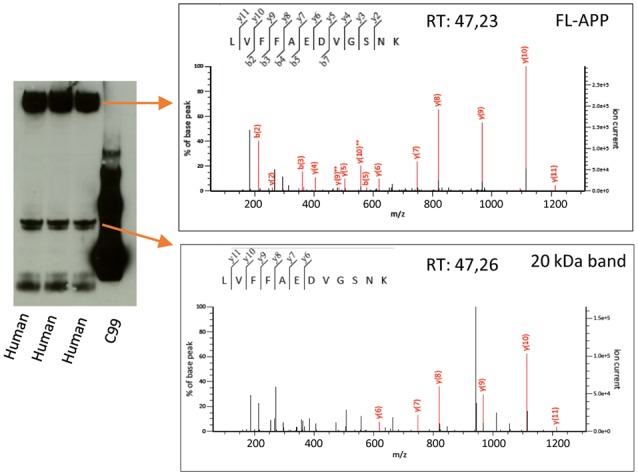
Mass spectrometry analysis of the 20 kDa band. Human AD brain homogenate was loaded on a 4%–12% gel and bands corresponding to the size of FL-APP or the 20 kDa band were cut out, in-gel digested with trypsin and subjected to liquid chromatography tandem mass spectrometry. The digested bands of both FL-APP (upper panel) and the 20 kDa band (lower panel) gave rise to spectra corresponding to the peptide LVFFAEDVGSNK of the APP sequence. Many other proteins were identified in the 20 kDa band to a higher score (Supplementary File S1).

**Figure 5:**
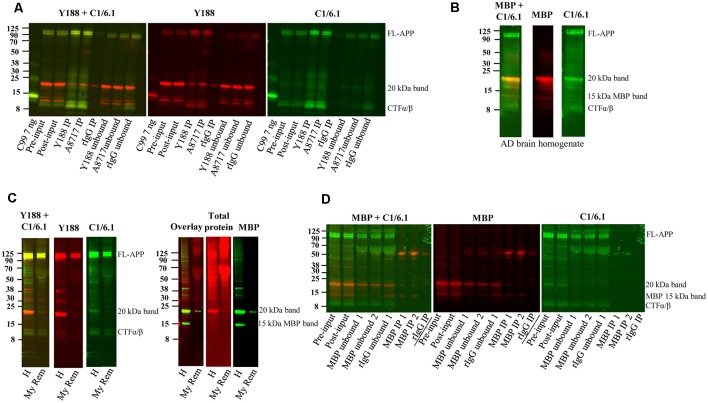
Immunoprecipitation of APP and depletion of myelin and myelin basic protein (MBP). **(A)** Western blot of immunoprecipitation of human AD brain homogenate using the Y188, the A8717 antibody or a rabbit immunoglobulin antibody (rIgG) as negative control. A recombinant C99-FLAG peptide was loaded as a size indicator. The 20 kDa band could not be immunoprecipitated to a higher level with the specific antibodies Y188 and A8717 than with the control rIgG, and the majority of the 20 kDa remained in the unbound fraction. On the contrary, full length (FL) APP and some of the lower molecular weight CTFs, were successfully immunoprecipitated. Fifteen percentage of the brain homogenate was collected before immunoprecipitation and loaded as input. This is a representative figure of eight experiments. **(B)** Western blotting of a human brain AD homogenate using the MBP and the C1/6.1 antibody shows overlap of the signal in the 20 kDa band. **(C)** A human brain AD homogenate was subjected to myelin removal beads (My Rem) or kept on ice (H), loaded on a gel and subjected to western blotting using Y188, C1/6.1 and MBP antibodies as well as a total protein stain. The loaded My Rem sample corresponded to 25 times more of the input than the loaded H sample. The 20 kDa band and the 15 kDa MBP band was depleted proportionally more than the FL-APP and APP-CTFα/β bands. **(D)** Immunodepletion of a human AD brain using an MBP antibody was performed using 2.5 μg (IP 1) or 5 μg (IP 2) MBP antibody or a rabbit IgG (rIgG) as negative control. Five percentage of input before (pre-input) and after (post-input) pre-clearing with beads only, as well of immunodepleted samples were loaded. Whereas the 15 kDa MBP band was efficiently and specifically depleted, the 20 kDa band detected by C1/6.1 was not depleted more by the MBP antibody than by the rIgG.

### The 20 kDa Band Is Also Present in Guinea Pig and Macaque Brain but Not in Mouse Brain or Human Embryonic Brain

Since the staining pattern of the different bands detected by APP antibodies was so different in human and rat brain (Figure 1), we decided to examine these bands in brain homogenates from a few more animal species. We found that the 20 kDa band was present also in brain homogenates from guinea pig, and to less extent in macaque, but not in mouse brain homogenates (Figure 2A). However, a faint band slightly above the prominent 20 kDa band was detected by C1/6.1 in all species. Again, the intensity ratios between 20 kDa band and the CTFα and -β bands were different depending on whether the Y188 or the C1/6.1 antibody was used for detection (Figure 2A).

To investigate whether the presence of the 20 kDa band is developmentally regulated, we next compared the expression pattern of APP-CTFs in brain homogenates prepared from embryo and adult (Figure 2B). Interestingly, the pattern of CTFs in brain homogenates from human fetus was clearly different from that in human adult brain, with undetectable levels of the 20 kDa band (Figure 2B). This pattern was further confirmed in three additional human fetus brain homogenates (data not shown). The pattern of CTFs in brain homogenates from mouse embryo was similar to that from adult mouse (Figure 2B).

In summary, the 20 kDa was present in adult brain from human, macaque and guinea pig, but was not present at detectable levels in adult brain from rat or mouse, nor in human fetal or mouse embryonic brain.

### Large Variation in Staining Intensity of the 20 kDa Band in Human Brain Samples

In order to determine whether there were any differences in the levels of the 20 kDa band between AD and control brain, we loaded equal amounts of homogenate from BA9 of the frontal cortex from 10 sporadic AD and 10 control subjects on 16% SDS-PAGE gels. Using both the Y188 and the C1/6.1 antibody for detection, we found that the 20 kDa was present in the majority of the brain homogenates from AD and control subjects (Figure 3, representative figure). The levels of the 20 kDa band varied considerably between individuals, and no significant differences could be detected between AD and control subjects. In addition, no obvious correlations could be found between levels of the 20 kDa band and parameters such as gender, age, post-mortem delay or storage time (data not shown). In order to normalize the levels to the total protein content, we also performed a total protein stain of the membrane. As in our initial experiments shown in Figure 1G, we noticed that a band that was intensively stained with the total protein stain overlapped in size with the 20 kDa band detected by the APP antibodies. Furthermore, the staining intensity of this band by the total protein stain correlated well with the intensity of the 20 kDa APP band.

Thus, the 20 kDa band was present in all human brain homogenates studied but that the levels varied considerably between samples and no significant differences in the levels of this CTF between AD and control cases. In addition, the band overlapped in both size and intensity with a major total protein band.

### Mass Spectrometry Confirms That the 20 kDa Band Contain APP but That Many Other Proteins Are Present in This Band as Well

The overlap of the 20 kDa APP band with a major total protein band raised our concern whether the antibodies studied cross-react or bind non-specifically to constituents of this band. In order to further elucidate the identity of this band, we therefore loaded human brain homogenate on a gel and cut out the bands corresponding to the size of full-length APP (as a positive control) and the 20 kDa band (as indicated by detection by western blotting using the Y188 antibody of parallel lanes of the same gel). The gel pieces were destained and alkylated, and in-gel digestion was performed. The digested band corresponding to full-length APP gave rise to a number of spectra of high quality whereof one corresponded to the LVFFAEDVGSNK peptide (Aβ 17–28, Figure 4, upper panel). This peptide was also identified in the digested 20 kDa band with a similar spectrum and retention time (Figure 4, lower panel), showing that this band indeed is partially derived from APP. However, the score of the APP-derived peptide was low and the digested gel piece also contained a large number of other proteins (Supplementary File S1). The protein with the highest score was MBP for which several of the major isoforms have the correct size of the digested band (17–21.5 kDa^1^). Other major hits included glutathione S-transferase P as well as different isoforms of tubulin beta and alpha. The peptides used for identifying the different tubulin isoforms were to a large degree overlapping.

### The 20 kDa Band Cannot be Efficiently Immunoprecipitated From Human Brain Using APP Antibodies and Is Depleted by Myelin Removal Beads but Not by Immunodepletion With an MBP Antibody

Next, we tested whether the 20 kDa band could be immunoprecipitated using the Y188 and the A8717 antibodies. To our surprise, immunoprecipitation of human AD brain homogenates with these antibodies immunoprecipitated only low amounts of the 20 kDa band and not significantly more than the IgG control (Figure 5A). In contrast, FL-APP, as well as the lower molecular weight APP-CTFs detected by the C1/6.1 antibody were very efficiently immunoprecipitated using either of the antibodies (Figure 5A). The large variation of the intensity of the 20 kDa APP-reactive band in human samples, and the correlation with the intensity of the same band visualized with a general protein stain, could potentially be explained by different amounts of white matter in these samples. In line with this, the levels of the 20 kDa APP band were undetectable in early human fetal stages (Figure 2B), where myelin is absent (Dubois et al., 2014). These observations, combined with the fact that MBP was the most abundant protein in the 20 kDa band, made us suspect that the labeling of the band was due to non-specific binding to myelin and/or cross-reaction of antibodies to the MBP protein. We, therefore, co-stained the immunoblots with the C1/6.1 and MBP antibodies. Several MBP isoforms between 14–21 kDa have been identified (Barbarese et al., 1977) and we detected several bands using the MBP antibody (Figure 5B). A major MBP-reactive band overlapped perfectly with the 20 kDa band detected with the C1/6.1 antibody (Figure 5B).

We also performed a myelin removal protocol using Myelin removal beads from Miltenyi Biotec. This product is developed to remove myelin from single-cell suspensions, not from tissue homogenates, and we experienced large loss of material during the procedure. However, proportionally more of the 20 kDa band was lost compared to the APP-FL and APP-CTFα and -β bands (Figure 5C). Thus, although we cannot be sure that this is due to the removal of myelin, the proteins represented by the 20 kDa band appear to bind more to the myelin removal beads (specifically or non-specifically) than the other APP bands do. An indication that myelin indeed was removed by this protocol was that both of the major MBP bands were also depleted. The intensity of the staining of the 20 kDa band with a total protein stain was also decreased after myelin depletion (Figure 5C).

To further elucidate whether the detection of the 20 kDa band by APP antibodies is due to cross-reactivity to MBP, we immunodepleted the homogenates from MBP using an MBP antibody. However, whereas an approximately 15 kDa band detected by the MBP antibody was efficiently depleted from the homogenates, and detected in the immunoprecipitates, the 20 kDa band detected by the C1/6.1 antibody was not specifically immunodepleted or immunoprecipitated using the MBP antibody. The 20 kDa band (as detected either by C1/6.1 or the MBP antibody) was, to a certain degree, immunodepleted both by the MBP antibody and the negative control rabbit IgG. In contrast, no differences in staining intensity could be observed before and after pre-clearing of the homogenates with beads only (Figure 5D).

Thus, both APP antibodies and the MBP antibody detects a 20 kDa band in human brain but the same antibodies cannot specifically immunoprecipitate this band, indicating that the detection is non-specific also for MBP. However, the protein(s) responsible for this staining can, to a certain degree, bind to any antibody-bead complexes (also negative control rabbit IgG) or to myelin removal beads but not to the magnetic beads alone.

### Several Other Antibodies Also Recognize a 20 kDa Band in Human Brain

**Figure 6:**
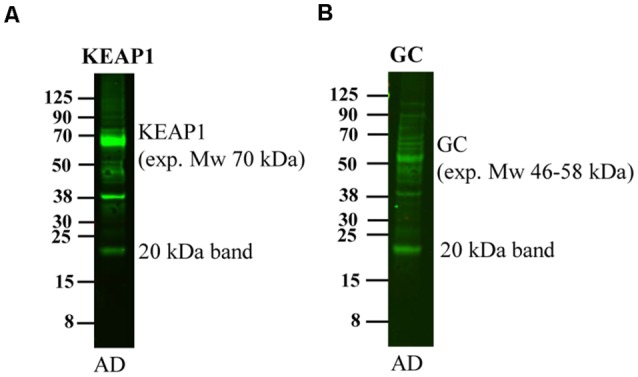
Detection of the 20 kDa band by other antibodies. An AD human brain homogenate was loaded on a 4%–12% gel and subjected to western blotting using antibodies directed to **(A)** Keap1 and **(B)** GC. These antibodies detect bands of their expected molecular weight of 70 and 46–58 kDa, respectively, but also detect a band of approximately 20 kDa.

Upon running western blotting experiments of human brain homogenates for other proteins in our laboratory, we noted that also these antibodies detected a band of approximately 20 kDa although the expected molecular weight was of a different size. These antibodies include Kelch-like ECH-associated protein 1 (Keap1, expected molecular weight 70 kDa, Figure 6A), and Vitamin D-binding protein (GC, expected molecular weight 46–58 kDa, Figure 6B). Thus, a multitude of antibodies, including APP antibodies, an MBP and other antibodies recognize this 20 kDa band, strongly indicating that the binding is at least partially non-specific.

## Discussion

Using western blotting to characterize the different APP-CTFs in brain homogenates, we here demonstrate that a band of approximately 20 kDa is abundant in human brain but not in rat or mouse brain. We performed a detailed investigation of this band in brain tissue from different species and developmental stages, AD patients and healthy controls, using a range of antibodies, mass spectrometry and immunoprecipitation.

Several studies have shown that bands that are detected by APP antibodies and migrate between 20 kDa and 30 kDa on SDS-PAGE gels are present in human brain (Estus et al., 1992; Haass et al., 1992; Tamaoka et al., 1992), but the identity of these bands has not been thoroughly investigated. However, recently Wang et al. (2015) identified an APP-band of similar size in human cell-lines and named it APP-CTFη. This band was further characterized by Willem et al. (2015) who also showed that subsequent cleavage of APP-CTFη by α-secretase resulted in a synaptotoxic Aη-α peptide. The detection of the APP-CTFη in mouse brain was specific since it was not detected in APP knock-out mice (Willem et al., 2015).

The 20 kDa band that we detect in human, guinea pig and macaque brain was much more prominent than any bands of similar size in mouse or rat brain, although we could detect a weak band of slightly higher molecular weight in mouse brain homogenates using the C1/6.1 antibody. In addition, the 20 kDa band was not present in human embryonic brain. Given the similarity in size between the 20 kDa band and the APP-CTFη band, we investigated whether the 20 kDa band could indeed be APP-CTFη. If so, the APP-CTFη levels would be much higher in human, guinea pig or macaque brain than in mouse or rat brain and thus reveal important species and developmental differences in APP processing. However, several facts indicate that the detection of the binding of APP antibodies to the 20 kDa band is partially non-specific:

(1) The binding of the Y188 and C1/6.1 to the 20 kDa band correlates both in molecular weight and intensity with a major band in a total protein stain. The total protein stain of this band is not likely to be due to staining of APP since APP was only detected with a low score in mass spectrometry analysis of this band. It should be noted, however, that APP is present in this band as well, albeit at low levels.
(2) The pattern of bands varied considerably depending on which APP antibody that was used. For example, the Y188 antibody binds proportionally more of the 20 kDa band than the APP-CTFα/β and APP-FL bands, despite the fact that the epitope of the antibodies should be present in all these fragments.
(3) Several other antibodies also detect the 20 kDa band although their expected molecular weight is not 20 kDa.
(4) It was not possible to specifically immunoprecipitate the 20 kDa band either with APP antibodies or with an MBP antibody even though other APP and MBP bands were efficiently immunoprecipitated.
(5) The 20 kDa band was proportionally more depleted than other APP fragments using a myelin removal protocol. Although this protocol is not designed for brain homogenates and we thus cannot exclude that proteins were non-specifically depleted, MBP was also depleted to a higher degree than APP-FL and APP-CTFα/β.

To conclude, these results indicate a non-specific binding of a multitude of antibodies to a 20 kDa band that also correlates to a band stained by a total protein stain. Regarding the proportional differences in binding capacity of different APP antibodies to the 20 kDa band compared to other APP fragments, as well as the inability to immunoprecipitate the 20 kDa band, we cannot totally rule out that this is due to a conformational change in the proposed APP-CTFη but we find this explanation quite unlikely.

The large variation of intensity of the 20 kDa band (detected by APP antibodies and a total protein stain) between different human brain samples could possibly be due to variation in white matter content. This note, together with the selective depletion of the 20 kDa band using a myelin removal protocol and the fact that MBP was the top hit in the MS analysis of the band, speak for that the binding was due to myelin. Using immunohistochemistry of human brain sections, the Y188 antibody also stained axonal fibers, more strongly than the C1/6.1 antibody (Jordá-Siquer et al. unpublished results), in line with the more prominent detection of the 20 kDa band with the Y188 than with the C1/6.1 antibody, further indicating binding to myelin. In addition, a previous study has shown non-specific immunohistochemical labeling of IgMs to myelin in human brain sections (Perentes and Rubinstein, 1986).

We could not, however, specifically immunodeplete the 20 kDa band detected by the C1/6.1 antibody using an MBP antibody, suggesting that the detection of this band is due to non-specific binding to a component in the 20 kDa band rather than cross-reactivity of these antibodies to MBP. The fact that we could non-specifically immunodeplete the C1/6.1-reactive 20 kDa band using a control rabbit IgG, as well as the detection of this band by a multitude of antibodies, further supports this conclusion. The myelin-removal beads technology also builds on immunodepletion but we cannot conclude whether this depletion is specific or not.

Although the major 20 kDa band, which is most prominently stained band by the Y188 antibody, is most probably to a large degree non-specific, we still detected an APP peptide in this band using in-gel digestion and mass spectrometry. Although the score of this peptide was low, it showed a similar spectrum and identical retention time as a peptide from the in-gel-digested FL-APP band, indicating that an APP-derived fragment of approximately 20 kDa indeed exists. In line with this, Willem et al. (2015) could detect the APP-CTFη in wild-type but not in APP knock-out mice, which strongly indicate the presence of a specific APP fragment of approximately 20 kDa in mouse brain. The levels of APP-CTFη in mouse brain and N2a cells were generally low but were increased upon BACE1 inhibition (Willem et al., 2015). In line with this, using the C1/6.1 antibody, we could detect a weak band migrating slightly above the major 20 kDa band in human brain. The fact that Willem et al. (2015) can detect low levels of APP-CTFη also using the Y188 antibody, whereas we cannot, is most probably due to slight differences in the detection limit between our protocols. Thus, this upper band might be a specific APP band, but in human brain this signal is hidden by the major potentially non-specific 20 kDa band. This fact makes it difficult to interpret results intended to rule out differences in levels of this band in different species, developmental stages or between AD and control brain.

García-Ayllón et al. (2017) reported on a 25 kDa fragment, presumably corresponding to APP-CTFη with increased levels in cerebrospinal fluid (CSF) of AD and Down syndrome patients compared to healthy controls. Since we do not know whether the major band detected by the total protein stain is present in CSF, we cannot determine whether the detection of the APP fragment in CSF is specific. In contrast to our results, García-Ayllón et al. (2017) succeeded in immunoprecipitating the 25 kDa band using Y188 or A8717 antibodies, which supports that the antibody binding to the band they detect is specific.

In summary, our results strongly indicate non-specific binding to a 20 kDa band in human brain by a multitude of APP and other antibodies. We call for precaution when analyzing proteins of a similar size in human brain. However, APP is present in this 20 kDa band and there is good evidence that a specific APP band of a similar size is present in mouse brain and possibly also in CSF.

## Data Availability Statement

The datasets generated for this study are available on request to the corresponding author.

## Ethics Statement

The studies involving human participants were reviewed and approved by the regional ethical review board of Stockholm (Regionala etikprövningsnämnden i Stockholm). The patients/participants provided their written informed consent to participate in this study. The animal study was reviewed and approved by the animal research ethical committee of southern Stockholm (Stockholm södra djurförsöksetiska nämnd) and Linköping ethical committee (Linköpings djurförsöksetiska nämnd).

## Author Contributions

JL, SF and HH planned the experiments. JL and HH performed the major part of the experiments. TK, TJ-S and MS performed the analysis of the different human brain samples. ES provided the human fetal tissue. MK performed the western blotting with Keap1 and GC antibodies. HH, JL, SF, LT, GB, ES and BW analyzed the data and contributed to the interpretation of the data. JL, SF and HH wrote the manuscript and all authors proof-read the manuscript.

## Conflict of Interest

The authors declare that the research was conducted in the absence of any commercial or financial relationships that could be construed as a potential conflict of interest.

## Footnotes

^1^www.uniprot.org/uniprot/P02686

## Abbreviations

Aβ: amyloid β-peptide
AD: Alzheimer disease
ADAM10: a disintegrin and metalloproteinase 10
AICD: amyloid precursor protein intracellular domain
APP: amyloid precursor protein
BA9: Brodmann area 9
BACE1: β-site amyloid precursor protein cleaving enzyme 1
CSF: cerebrospinal fluid
CTF: C-terminal fragment
DTT: Dithiothreitol
EDTA: Ethylenediaminetetraacetic acid
FL-APP: full length APP
GC: Vitamin D binding protein
H: homogenate
HRP: horseradish peroxidase
KEAP1: Kelch-like ECH-associated protein 1
PMD: post-mortem delay
PVDF: polyvinylidene difluoride
RIPA: radio immunoprecipitation assay
sAPP: soluble amyloid precursor protein
SDS-PAGE: sodium dodecyl sulfate polyacrylamide gel electrophoresis.
